# The relationship between baseline nutritional status with subsequent parenteral nutrition and clinical outcomes in cancer patients undergoing hyperthermic intraperitoneal chemotherapy

**DOI:** 10.1186/1475-2891-12-118

**Published:** 2013-08-14

**Authors:** Pankaj G Vashi, Digant Gupta, Carolyn A Lammersfeld, Donald P Braun, Brenten Popiel, Subhasis Misra, Komen C Brown

**Affiliations:** 1 Cancer Treatment Centers of America® (CTCA) at Midwestern Regional Medical Center, 2520 Elisha Avenue, Zion, IL 60099, USA; 2 Cancer Treatment Centers of America®, 1336 Basswood Road, Schaumburg, IL 60173, USA

## Abstract

**Background:**

The combination of cytoreductive surgery (CRS) and hyperthermic intraperitoneal chemotherapy (HIPEC) is a promising treatment option for selected patients with peritoneal carcinomatosis. This retrospective study investigated the relationship between baseline nutritional assessment with subsequent parenteral nutritional (PN) and clinical outcomes in cancer patients undergoing CRS and HIPEC.

**Methods:**

A consecutive series of 60 patients undergoing CRS and HIPEC at our institution between January 2009 and May 2011. Subjective Global Assessment (SGA) was used to assess nutritional status. Patients were classified preoperatively as: well nourished (SGA-A), mildly-moderately malnourished (SGA-B), and severely malnourished (SGA-C). For PN, patients were divided into 2 groups: those who received PN (PN+) and those who did not receive PN (PN-). The primary outcomes of interest were length of stay (LOS), postoperative complications, ECOG performance status (PS) and survival. LOS was calculated as the number of days in the hospital post surgery. Performance status was measured on a scale of 0-4. Survival was calculated from the date of first visit to the date of death/last contact.

**Results:**

Of 60 patients, 19 were males and 41 females. The mean age at presentation was 50.3 years. The most common cancer types were colorectal (n = 24) and gynecologic (n = 19) with the majority of patients (n = 47) treated previously before coming to our institution. 33 patients were SGA-A, 22 SGA-B and 5 SGA-C prior to surgery. Of a total of 60 patients, 31 received PN. Mean LOS for the entire cohort was 16.2 days (SD = 9.8). Mean LOS for preoperative SGA-A, SGA-B and SGA-C were 15.0, 15.2 and 27.8 days respectively (ANOVA p = 0.02). Overall incidence of complications was 26.7% (16/60). Complications were recorded in 9 of 33 (27.3%) preoperative SGA-A patients and 7 of 27 (25.9%) SGA-B + C patients (p = 0.91). The median overall survival was 17.5 months (95% CI = 13.0 to 22.1 months). Median survival for preoperative SGA-A and SGA-B + C cohorts was 22.4 and 10.4 months respectively (p = 0.006).

**Conclusions:**

The preoperative SGA predicts LOS and survival in cancer patients undergoing HIPEC. Future randomized clinical trials in this patient population should investigate the systematic provision of PN to all malnourished patients in the preoperative period for a minimum of 7-10 days with the continuation of PN in the postoperative period.

## Background

Cytoreductive surgery (CRS) and hyperthermic intraperitoneal chemotherapy (HIPEC) as a combined treatment modality is a promising therapeutic option for selected patients with peritoneal carcinomatosis arising from different malignancies such as colorectal cancer, gastric cancer, ovarian cancer or peritoneal mesotheliomas [1]. Numerous studies with different levels of evidence have demonstrated a survival advantage in patients treated with CRS and HIPEC as compared to those treated with systemic chemotherapy alone [2-7]. However, the survival advantage often comes at the expense of significant morbidity, which has been reported at a rate of 25% to 41% and can primarily be divided into surgery-related and chemotherapy-related complications. Common surgery-related complications are postoperative ileus, anastomotic leakage, wound infection, bleeding, intra-abdominal abscess, deep vein thrombosis and lung embolism while the different cytostatic agents used for HIPEC can lead to leucopenia, anemia, thrombopenia, heart, liver or renal toxicity and other side effects [1,8]. These morbidities are often associated with a negative impact on patients’ nutritional status and quality of life [9-11].

While there are no data available on the prevalence of malnutrition in patients with peritoneal carcinomatosis, malnutrition is observed in up to 67% of patients with ovarian cancer [12] and up to 80% of patients with advanced colorectal cancer [13], the two most common cancers associated with the development of peritoneal carcinomatosis. Moreover, there is enough evidence in the literature documenting the association of malnutrition with longer hospital stay, reduced response, increased complications to anticancer therapy, increased overall cost of care, and poor survival and quality of life [13-19]. As a result, it has been recommended that nutritional screening be performed in all peritoneal carcinomatosis patients who are potential candidates for CRS and HIPEC, while a more in-depth nutritional assessment followed by adequate nutritional intervention be considered in malnourished patients [10]. However, there are no data available in the literature on the role of nutritional assessment and support in peritoneal carcinomatosis patients undergoing CRS and HIPEC.

The aim of this study was to investigate the relationship between baseline nutritional assessment with subsequent parenteral nutritional (PN) and clinical outcomes in cancer patients undergoing CRS and HIPEC.

## Methods

### Study population

This was a retrospective study performed on a consecutive case series of 60 cancer patients treated with R0/R1 CRS and HIPEC at Cancer Treatment Centers of America® (CTCA) at Midwestern Regional Medical Center (MRMC) between January 2009 and May 2011. Only patients with a histologically confirmed diagnosis of cancer were included. The study did not restrict patients with respect to treatment history, tumor histology or stage. This study was approved by the Institutional Review Board at Midwestern Regional Medical Center (MRMC).

### CRS and HIPEC

After opening the abdomen, the presence of macroscopic tumor deposits was recorded in 13 abdominopelvic regions namely central abdomen, right upper quadrant, epigastrium, left upper quadrant, left flank, left lower quadrant, pelvis, right lower quadrant, right flank, upper jejunum, lower jejunum, upper ileum and lower ileum [20]. This leads to the calculation of a peritoneal carcinomatosis index (PCI) score [21] which standardizes the reporting of tumor burden in patients with carcinomatosis. The PCI is an assessment combining lesion size (0–3) with tumor distribution to quantify the extent of disease as a numeric score. The score was calculated at the time of surgical exploration of the abdomen and pelvis. The PCI is of great value in the process of deciding between a surgically aggressive complete cytoreduction and a palliative debulking procedure [21].

After CRS, the patient was then prepared for HIPEC. Catheters were placed, one in each subdiaphragmatic space for outflow catheter and connected to a Y-connector and brought out through the midline incision. The inflow catheter was placed down in the pelvis and was similarly connected to a Y-connector, and then brought out in the cephalad portion of the midline incision. Temperature probes were placed in each anterior abdominal wall, and also brought out through the midline incision. The midline incision was closed by using a running #1 Prolene in a baseball stitch fashion. The catheters were then hooked up to the perfusion circuit. The abdominal cavity was instilled with 3 liters of crystalloid solution and perfused with warming, until a temperature of 41 degrees Celsius was achieved. At this time, assessment was done to make sure there were no leaks noted from the abdominal cavity. 30 mg of Mitomycin-C was then added into the perfusion circuit for perfusion of the abdomen. The patient was perfused for a total of 60 minutes with an average flow of approximately to 1700 to 2000 mL per minute. The temperature was between 41 to 42 degrees Celsius, as monitored by the temperature probe with an inflow temperature of 43 degrees Celsius. After these 60 minutes of perfusion, an additional 10 mg of Mitomycin-C was added into the perfusion circuit and the patient was perfused for an additional 30 minutes. At the completion of the 90 minute perfusion, the perfusate was flushed and the abdomen was additionally flushed with 3 liters of crystalloid solution, following which the flushed solution was evacuated from the abdomen. The abdomen was then reopened and the remaining portion of the surgery was completed.

### Nutritional status assessment

All patients in this study were scheduled for a consultation with a dietitian. Prior to each consultation, a dietitian reviewed the patient’s history from the medical record and verified the patient’s current weight. Subjective Global Assessment (SGA) was used to assess nutritional status. The SGA is a clinical technique that combines data from subjective and objective aspects of medical history (weight change, dietary intake change, gastrointestinal symptoms, and changes in functional capacity) and physical examination (loss of subcutaneous fat, muscle wasting, ankle or sacral edema and ascites). After evaluation, patients are categorized into three distinct classes of nutritional status; well nourished (SGA-A), moderately malnourished (SGA-B) and severely malnourished (SGA-C) as described by Detsky et al [22]. The SGA has been validated in a number of diverse patient populations, including cancer patients [23-27]. It has also been correlated with a number of objective nutritional assessment indicators, morbidity, mortality, and QoL measures [28-31]. At the subjects’ first visit, measurement of height and weight were performed. The subjects wore light clothing and no shoes. BMI was calculated as weight (kg) / squared height (m^2^).

### Parenteral nutrition

PN was administered as per American Society of Parenteral and Enteral Nutrition (ASPEN) guidelines [32]. Total daily calories given were 25-30 kcal/kg for BMI <30 and 22-25 kcal/kg of ideal body weight if BMI >30. Proteins were given at 2 g/kg for BMI < 30 and 2.5 g/kg of ideal body weight if BMI > 30. Calories from lipids were limited to < 30% of total daily requirement. The decision to consider PN in our patients depended on two major factors – preoperative SGA status and presence or absence of bowel obstruction. PN was recommended for all patients with preoperative SGA-C. All patients with bowel obstructive symptoms, irrespective of their SGA status were considered for PN.

The patients were monitored closely for their nutritional status and recovery of the gastrointestinal functions after CRS/ HIPEC. PN was initiated in those patients who had delayed gastric function recovery or who had multiple bowel resections. Nutrition and Metabolic Support Team (NSMT) followed all the patients and initiated PN within 7 days after the surgery when indicated. Many patients were discharged with PN which was discontinued only when adequate oral intake was established.

### Statistical analysis

Nutritional status (as measured by SGA) and PN were the primary independent variables of interest. SGA was used as a categorical variable (SGA-A, SGA-B and SGA-C). For PN, patients were divided into 2 groups: those who received PN (PN+) and those who did not receive PN (PN-).

The primary outcomes of interest were length of stay (LOS), postoperative complications, ECOG performance status and survival. LOS was calculated as the number of days patients stayed in the hospital post surgery. Postoperative complications were judged by the attending surgical oncologist and gastroenterologist and defined as any clinical event not typically seen in patients who receive HIPEC. Performance status was measured on a scale of 0-4. Survival was defined as the time interval between the date of first visit to our hospital and the date of patient’s death from any cause or the date of last contact/last known to be alive. Patients alive at the time of this analysis (June 2013) were considered censored for the purpose of this analysis.

Mean LOS was compared across the 3 SGA groups and 2 PN groups using Analysis of Variance (ANOVA) and independent sample t-test respectively. Incidence of complications was compared across the SGA and PN groups using chi-square tests. The survival was estimated using the Kaplan-Meier method and tested with the log-rank test across the SGA and PN groups. All data were analyzed using IBM SPSS version 20.0 (IBM, Armonk, NY, USA). All analyses were two-tailed, and a difference was considered to be statistically significant if the p value was less than or equal to 0.05.

## Results

### Baseline characteristics

Table 1 describes the baseline characteristics of our patient cohort. Of a total of 60 patients, 31 received PN. Of those 31 patients, 23 received postoperative PN while 8 received PN both pre- and postoperatively. The average duration of preoperative PN was 5.1 days while that of postoperative PN was 27.4 days. Thirteen patients were analytic (newly diagnosed and treated at our institution) while 47 patients were non-analytic (previously treated elsewhere prior to coming to our institution). At the time of this analysis (June 2013), 38 (63.3%) patients had expired. The mean follow-up time duration was 15.1 months with a range of 1.9 to 37.8 months.

**Table 1:** Baseline patient characteristics (N = 60)

**Characteristic**	**Categories**	**Number**	**Percent (%)**
Gender	Males	19	31.7
	Females	41	68.3
Age at presentation (years)	Mean	50.3	
	Median	51.8	
	Range	21.4 – 69.1	
Cancer site	Colorectal	24	40
	Gynecologic	19	31.7
	Appendix	8	13.3
	Peritoneal	5	8.3
	Others (omentum, small intestine, stomach, unknown)	4	6.7
Treatment history	Analytic	13	21.7
	Non-analytic	47	78.3
Preoperative SGA	A	33	55
	B	22	36.7
	C	5	8.3
PN	No	29	48.3
	Yes	31	51.7

Table 2 compares the patient characteristics between the PN + and PN- groups. As compared to the PN- group, the PN + group had a greater proportion of male patients as well as a greater proportion of patients with previously treated disease, gynecologic cancers and malnourished status prior to surgery. There were no statistically significant differences in the mean PCI score and the length of CRS and HIPEC procedure between the 2 PN groups. There were a total of 4 peritonectomies, 1 in the PN- group and 3 in the PN + group (p = 0.33). The total number of anastomoses/resections in the PN- and PN + groups was 30 and 35 respectively. The median was 1 in both the groups (p = 0.93).

**Table 2:** Baseline characteristics of PN+ and PN- groups

**Characteristic**	**PN- (n = 29)**	**PN+ (n = 31)**	**P-value**
Gender			
Males	5 (17.2%)	14 (45.2%)	0.02
Females	24 (82.8%)	17 (54.8%)	
Mean age at presentation (years)	50.3	50.3	0.99
Cancer site			
Colorectal	11 (37.9%)	13 (41.9%)	0.91
Gynecologic	8 (27.5%)	11 (35.4%)	
Appendix	5 (17.2%)	3 (9.7%)	
Peritoneal	3 (10.3%)	2 (6.5%)	
Others	2 (6.9%)	2 (6.5%)	
Treatment history			
Analytic	8 (27.6%)	5 (16.1%)	0.28
Non-analytic	21 (72.4%)	26 (83.9%)	
Preoperative SGA			
A	20 (69%)	13 (41.9%)	0.10
B	7 (24.1%)	15 (48.4%)	
C	2 (6.9%)	3 (9.7%)	
Mean PCI score	17.2	20.4	0.17
Length of CRS and HIPEC (minutes)	662	673	0.82

Values in parenthesis are column percentages.

### LOS

Mean LOS for the entire study population was 16.2 days (standard deviation = 9.8). The mean LOS for patients in the pre-operative SGA-C group was significantly longer than the mean LOS for those in the SGA-A and SGA-B groups as shown in Table 3. Similarly, the mean LOS for patients in the PN + group was significantly longer than the mean LOS for patients in the PN- group.

**Table 3:** Mean LOS across preoperative SGA and PN groups

**Independent variable**	**Mean LOS (days)**	**P-value**
**Preoperative SGA**		
• Well nourished (n = 33)	15.0	ANOVA
• Moderately malnourished (n = 22)	15.2	p = 0.02
• Severely malnourished (n = 5)	27.8	
**PN**		
• No (n = 29)	12.7	2 sample t-test
• Yes (n = 31)	19.4	p = 0.007

### Perioperative morbidity

There were a total of 4 readmissions within 30 days of discharge, 1 in the PN- group and 3 in the PN + group (p = 0.33). The reasons for readmissions were pelvic abscess and wound infection with fistula in the PN- group and copious discharge per rectum, abdominal pain, and wound dehiscence in the PN + group. Only one patient expired within 30 days of surgery in the PN + group, whereas there were no deaths within 30 days of surgery in the PN- group.

### Complications

Overall incidence of complications was 26.7% (16/60). Nine patients had 1 complication, two patients had 2, 3 and 4 complications each and 1 patient had 10 complications. The number of complications was correlated to LOS (Pearson r = 0.3, p = 0.03). Complications were recorded in 9 of 33 (27.3%) pre-operative SGA-A patients and 7 of 27 (25.9%) SGA-B + C patients (chi-square p = 0.91). Similarly, complications were recorded in 4 of 29 (13.8%) PN- patients and 12 of 31 (38.7%) PN + patients (chi-square p = 0.03). Most common were wound-related complications and sepsis.

### Survival

**Figure 1:**
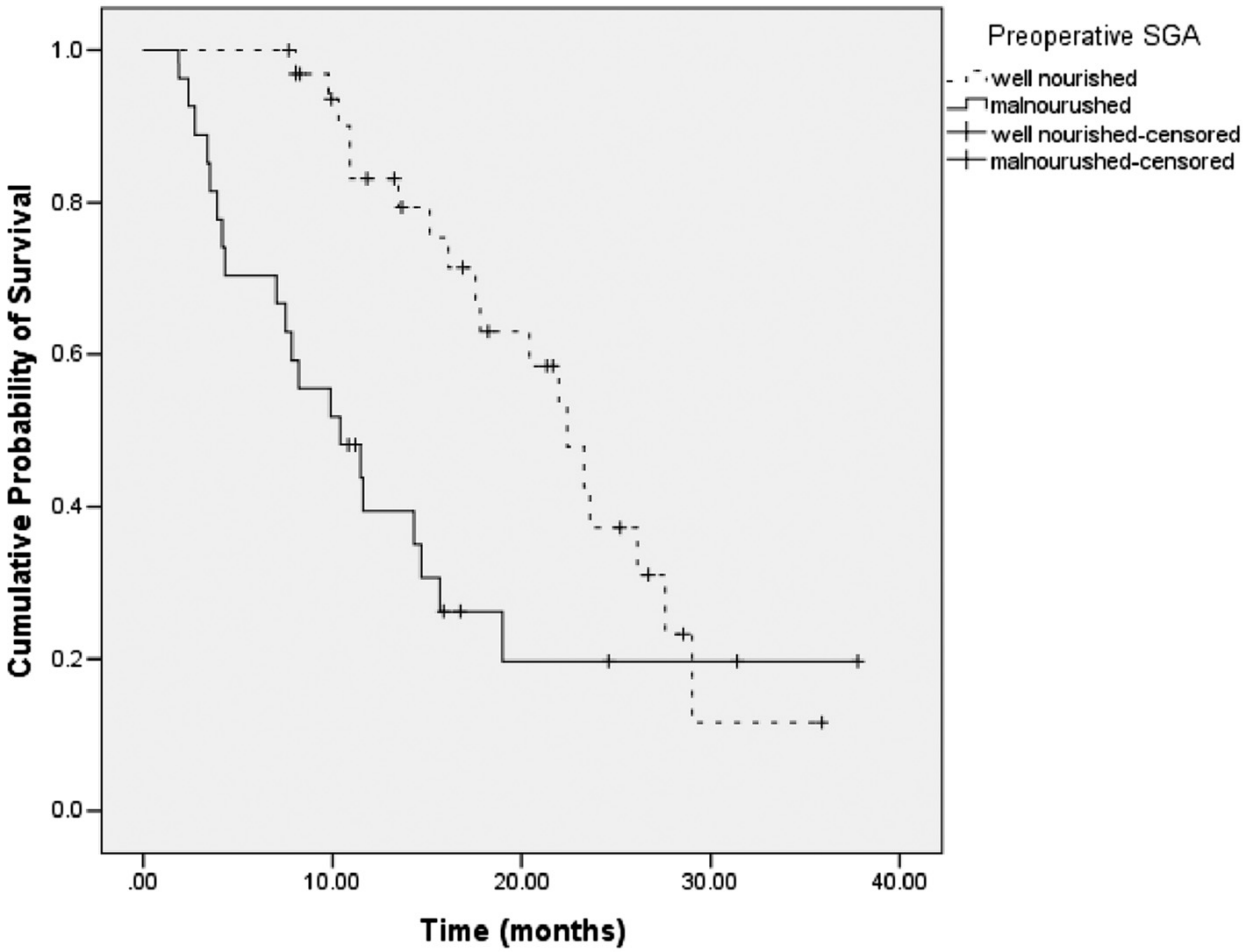
**Survival stratified by 2 categories of preoperative SGA.** Each drop in a probability curve indicates one or more events in that group. Vertical lines indicate censored patients, i.e., those who reached the end of their follow-up without experiencing death.

The median overall survival for the entire patient cohort was 17.5 months (95% CI = 13.0 to 22.1 months). Figure 1 shows the survival curves for the 2 categories of preoperative SGA. Well nourished patients had a median survival of 22.4 months (95% CI: 18.7 to 26.1), while malnourished patients had a median survival of 10.4 months (95% CI: 5.2 to 15.7); the difference being statistically significant (log rank p = 0.006).

**Figure 2:**
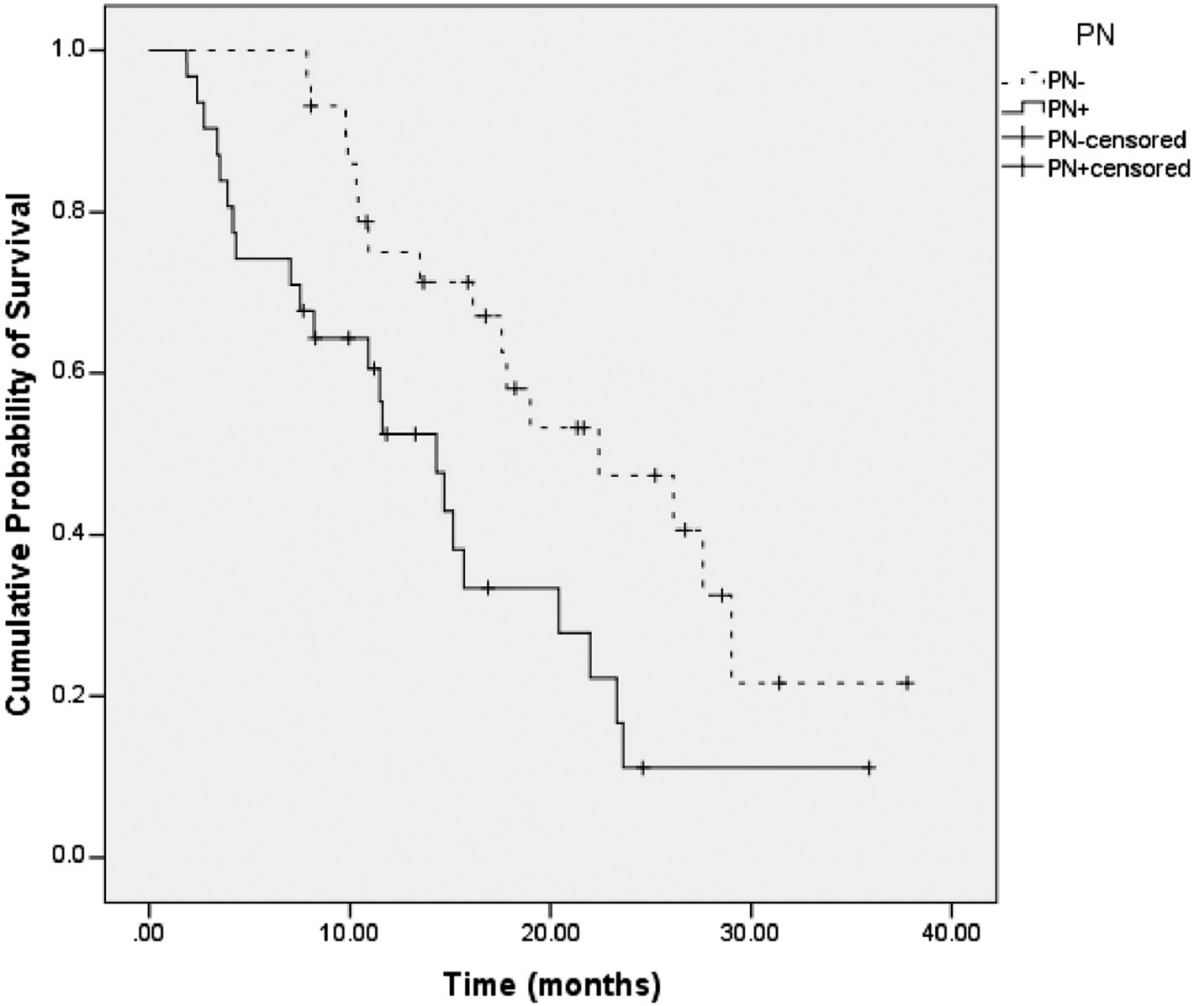
**Survival stratified by 2 PN groups.** Each drop in a probability curve indicates one or more events in that group. Vertical lines indicate censored patients, i.e., those who reached the end of their follow-up without experiencing death.

Figure 2 shows the survival curves for the 2 PN groups. Patients in the PN + group had a median survival of 14.3 months (95% CI: 9.9 to 18.8), while patients in the PN- group had a median survival of 22.4 months (95% CI: 12.6 to 32.2); the difference being statistically significant (log rank p = 0.01).

### Subgroup analysis by SGA

We conducted a subgroup analysis to compare the outcomes (LOS, survival and post-operative complications) between the PN + and PN- groups in preoperative SGA-A patients only. No statistically significant differences were found between the two PN groups with regard to any of the outcomes.

### SGA and ECOG status

For the entire population (n = 60), the mean preoperative and postoperative SGA scores were 4.4 and 5.7 respectively, while the corresponding PS scores were 1.3 and 1.6 respectively, p < 0.05 for both. In the PN + group (n = 31), the mean preoperative and postoperative SGA (5.1 and 6.6) and PS (1.5 and 1.9) scores were not significantly different; p > 0.05 for both. In the PN- group (n = 29), preoperative and postoperative SGA scores were not significantly different but postoperative PS score (1.4) was significantly worse than the preoperative PS score (1.1); p = 0.001.

## Discussion

Cancer patients are particularly susceptible to nutritional depletion due to the combined effects of the malignant disease and its treatment. CRS followed by HIPEC is a major surgical procedure that can further accentuate the risk of nutritional depletion in patients with peritoneal carcinomatosis [10,11,33]. As a result, timely nutritional assessment and intervention in this patient population might be critical to achieving optimal clinical outcomes including LOS, cost, quality of life, survival and ability to tolerate treatment. In this retrospective study of the first 60 patients at our institution who had CRS and HIPEC for peritoneal carcinomatosis, we investigated the relationship between baseline nutritional status and clinical outcomes. We also conducted a preliminary analysis of clinical outcomes as a function of parenteral nutrition.

There are 2 key findings of our study. Baseline nutritional status, as evaluated using SGA, was predictive of patient LOS. This finding is consistent with the existing literature in this area. A recently published systematic review (based on a total of 21 studies ) on the role of nutritional status in predicting LOS in cancer concluded that validated nutritional tools such as SGA are good predictors of LOS in gastrointestinal cancers requiring surgery [34]. Since CRS and HIPEC are associated with significant morbidity which can potentially increase the LOS, it is prudent to provide nutrition support during the perioperative period in these individuals. It makes sense to implement the ASPEN guidelines [32] for these patients, which include nutritional screening, assessment, and intervention as appropriate. Correcting malnutrition may decrease the LOS and perhaps even reduce the rate of hospital readmissions in this population. Consistent with the vast body of existing literature in this area [14,15], we also found that baseline nutritional status, as evaluated using SGA, was a significant predictor of survival in this patient population.

The LOS in patients who received PN was longer than in patients who did not receive PN. Similarly, the survival in the PN + group was shorter than in the PN- group. This was expected considering the patients who did not receive PN had regained gastrointestinal functions within 7 days of surgery and were better nourished than patients who received PN. Given the lack of comparability between the PN + and PN- groups, no conclusions related to causation can be drawn from these findings.

The peritoneal malignancy program at our institution was started by a dedicated team of physicians, nurses and surgical staff and led by a surgical oncologist with special training in CRS and HIPEC. Our experience with the first 60 patient in this study has helped us develop processes for nutritional evaluation and interventions with oral, parenteral or enteral nutrition in the pre- and post-operative periods. Guidelines established by ASPEN for nutritional support in surgical patients were used for all patients [32]. Although the role of nutritional support has not been studied in patients undergoing CRS and HIPEC, the benefits of perioperative nutrition have been well-established for other planned major abdominal surgeries [35]. As part of future research in this area, the role of additional pre-operative enteral nutrition to enhance nutritional status in eligible cohorts could be examined.

Some limitations of this study need to be acknowledged. Our study, because of its retrospective nature, relies on data not collected to test a specific hypothesis. A majority of our patients had advanced stage disease at presentation and had failed primary treatment elsewhere before coming to our hospital. As a result, we acknowledge that our findings may not be applicable to newly-diagnosed patients with peritoneal carcinomatosis, an issue that needs to be tested in suitable patient populations. Our retrospective study was not designed to investigate a causative relationship between PN and clinical outcomes. This is evident by the fact that our PN + and PN- groups were substantially different from each other with regard to the baseline clinical and demographic characteristics. As compared to the PN- group, the PN + group had a greater proportion of male patients as well as a greater proportion of patients with previously treated disease, gynecologic cancers and malnourished status prior to surgery. As a result, no definitive conclusions can be made regarding the role of parenteral nutrition in improving clinical outcomes in this patient population. Prospective randomized clinical trials are needed to this effect. Our study had a relatively small sample size of 60 patients. Finally, an overall complication rate of 26.7% could be an underestimate owing to the retrospective nature of this study and the lack of complication grading criteria. Despite these limitations, to the best of our knowledge, this is the first study to evaluate the prognostic significance of nutritional assessment in peritoneal carcinomatosis patients undergoing CRS and HIPEC.

## Conclusions

The preoperative SGA predicts LOS and survival in cancer patients undergoing HIPEC. Future randomized clinical trials in this patient population should investigate the systematic provision of PN to all malnourished patients in the preoperative period for a minimum of 7-10 days with the continuation of PN in the postoperative period.

## Competing interests

The authors declare that they have no competing interests.

## Authors’ contributions

PGV participated in concept, design, data collection, data interpretation and writing. DG participated in data collection, data analysis, data interpretation and writing. CAL and BP participated in data collection, data interpretation and writing. DPB participated in data interpretation, writing and general oversight of the study. SM and KCB participated in concept, design, data interpretation and writing. All authors read and approved the final manuscript.
